# Prognosis and chemotherapy drug sensitivity in liver hepatocellular carcinoma through a disulfidptosis-related lncRNA signature

**DOI:** 10.1038/s41598-024-57954-7

**Published:** 2024-03-26

**Authors:** Chao Chen, Chaoyang Wang, Yi Li, Shanshan Jiang, Ningjun Yu, Guofeng Zhou

**Affiliations:** 1 Department of Radiology, Union Hospital, Tongji Medical College, Huazhong University of Science and Technology, Wuhan, 430022 Hubei China; 2 Department of Radiology, Sichuan Science Hospital, Mianyang, 621022 Sichuan China

**Keywords:** Disulfidptosis, Long noncoding RNA, HCC, Prognostic signature, Drug sensitivity, Computational biology and bioinformatics, Cancer

## Abstract

Disulfidptosis, a new type of regulated cell death associated with the actin cytoskeleton, provides a new therapeutic tool for cancers. The direct relationship between disulfidptosis-related lncRNAs(DRLs) in liver hepatocellular carcinoma(HCC) remains unclear. We acquired transcriptomic data, corresponding clinical data, and tumor mutation data of HCC from the TCGA database. First of all, DRLs were determined through correlation analysis. Then, a prognostic model containing six DRLs was created by adopting univariate Cox regression, LASSO algorithm and multivariate Cox regression analysis. Based on the model, 424 HCC patients were divided into high- and low-risk groups. Next, we structured ROC curves and PCA through combining the model and clinical data. Enrichment analysis and immune infiltration analysis were adopted to further explore the relationship between the model and prognosis. In addition, we explored the relationship between the model and tumor mutation burden (TMB). There were significant differences between high- and low- risk groups, and patients in the high-risk group showed poor prognosis. Enrichment analysis suggested that metabolic progress was obviously different between the two groups. According to the analysis of immune infiltration, there were several differences in immune cells, function, and checkpoints. Patients with high-risk and high TMB demonstrated the least favorable prognosis. The two risk groups both manifested visiblly in chemotherapy drug sensitivity. To sum up, we set up a DRL-based signature and that may provide a predictable value for the prognosis and use of chemotherapy drugs for HCC patients.

## Introduction

Liver cancer is one of the most malignant cancers in the world and has an extremely poor prognosis. According to recent global cancer statistics, liver cancer ranks third in terms of cancer associated mortality, although it ranks only sixth in cancer related incidence^1^. Moreover, the number of deaths from liver cancer ranks second among all cancers in China^2^. Among all pathological types, HCC is the most predominant type. Surgical resection is the main treatment method for patients with early-stage disease. Without obvious symptoms, patients often become unsuitable for surgical treatment when first diagnosed with liver cancer. While interventional therapy (including transcatheter arterial chemoembolization (TACE), microwave ablation, and particle implantation), targeted therapy, and immunotherapy have been proven effective for most patients with advanced HCC, such methods can only prolong the survival period and the prognosis remains unsatisfactory in most cases^3^. Even though it is widely known that hepatitis B virus (HBV) is associated with HCC, hepatocarcinogenesis is a complex process in which many signal pathway transductions are altered^4^. Mutation is the most common molecular pathogenesis, which may lead to different types of treatments^5^.

Disulfidptosis, a novel metabolic-related regulated cell death (RCD), is of peculiar value in the area of cancer metabolic therapy^6^. High expression of solute carrier family 7 member 11 (SLC7A11) could result in the accumulation of cysteine which would contribute to disulfide stress in cell metabolism^7^. When anomalous disulfide bonds form between actin cytoskeleton proteins, a lack of glucose and high expression of SLC7A11 would cause the excessive accumulation of disulfides, which could eventually lead to cell shrinkage and death^8^. It is worth mentioning that disulfidptosis would not be suppressed by diverse kinds of cell death inhibitors, even though genes related to cell death had been knocked out^9^. Based on this discovery, disulfidptosis may serve as a novel measure for tumor treatment. A small portion of regulatory mechanisms had been identified recently^9^. However, further exploration and identification of disulfidptosis are urgently needed and it is necessary to discover the underlying mechanism.

Long noncoding RNAs (lncRNAs), a group of non-protein-coding transcripts with at least 200 nucleotides, are now well recognized to regulate energy metabolism in cancer^10^. An increasing number of studies have revealed that lncRNAs are closely associated with the occurrence and development of tumors and have a dual effect on promoting and inhibiting cancer^11^. For instance, downregulating the expression of LINC01060 could inhibit tumor growth and metastasis in osteosarcoma through PI3K/AKT signaling^12^. Similarly, silencing HOTAIR significantly reduces the migration ability of cells and induces cell apoptosis in cervical cancer^13^. Liu et al. found that LIN01268 can promote pancreatic cancer through PI3K/AKT aixs^14^. In human hepatocellular carcinoma, Guo et al. found the high expression of LNC02870 which could result in migration and invasion of cancer^15^. TPA has been shown to promote the progression of breast cancer via the TGF-β signaling pathway^16^.

In this study, we extracted transcriptome data of patients with HCC from The Cancer Genome Atlas (TCGA) database. We identified 746 lncRNAs that were significantly correlated with disulfdptosis-related genes (|Cor|> 0.4 and *P* < 0.001). Then, we constructed a prognostic model containing 6 disulfdptosis-related lncRNAs, including AC007406.2, AL049840.3, AC138696.2, MKLN1-AS, AC069307.1 and TMCC1-AS1. Subsequently, we investigated the predictive ability through combining the risk score and clinical data. We also evaluated the biological properties, immune infiltration, TMB, and drug responsiveness of the model. The aim of this study was to offer new perspectives and approaches for clinical immunotherapy and potential chemotherapeutic drugs for HCC.

## Materials and methods

### Data collection

Transcriptomic data, corresponding clinical data and simple nucleotide variation(SNV) of patients with HCC (374 tumor tissues and 50 normal tissues) from The Cancer Genome Atlas (TCGA) database (https://www.cancer.gov/). The Perl programming language (version 5.30.0.1) was used to arrange data including transcriptomic and clinical information for subsequent analysis.

### Collection of the disulfdptosis-related genes and lncRNAs

The disulfdptosis-related genes list was gathered from the previous study of Liu and their colleagues^17^. Correlation analysis was used to assess the relationship between disulfdptosis-related genes and lncRNAs and Sankey diagram was plotted to visualize the results. The standards for the correaltion are |Cor|> 0.4 and *P* < 0.001.

### Construction of the prognosis model

All patients were randomly separated into a training cohort and a test cohort at a ratio of 1:1. Univariate regression analysis was applied to screen genes associated with prognosis (*P* < 0.05). The least absolute shrinkage and selection operator (LASSO) and multivariate Cox regression were used to further filter the lncRNAs for model establishment. The final formula of the model was as follows.

$${\text{Risk Score}}\left( {{\text{patient}}} \right) = \Sigma {\text{iCoefficient}}({\text{lncRNA}}_{{\text{i}}} ) \, \times {\text{ Expression}}\left( {{\text{lncRNA}}_{{\text{i}}} } \right).$$

### Characterization of the model

Datas were classified by the median risk score threshold in train group as high- or low-risk groups. To estimate the predictive value of the model, we used a series of methods. The R packages "survminer" and "survival" were utilized to depict overall survival (OS) and progression-free survival (PFS). Scatter plots, risk curves, and heatmaps were portrayed to valuate the differences between high- and low-risk groups. Such methods were taken in the training cohort, test cohort and TCGA cohort.

### Validation of the prognostic model

Univariate and multivariate Cox regression analyses were implemented to confirm the independence of the prognostic model with other clinical characteristics (age, gender, grade stage, Alpha-fetoprotein (AFP), Prothrombin Time (PT), Total Bilirubin (TB), Albumin (Alb), Platelet). Receiver operating characteristic (ROC) curves were drawn to evaluate the sensitivity and specificity of the prognostic model compared with other clinical characteristics. In addition, an external cohort from GEO database(https://www.ncbi.nlm.nih.gov/geo/) was used to validate the stability and generalization ability of the model.

### PCA and nomogram construction

Principal component analysis (PCA) was applied to investigate whether the risk score could be a meaningful signature to classify patients. A nomogram was created through integrating the risk score and other prognostic characteristics to predict the 1-, 3- and 5-year OS of HCC patients by using the R package "regplot". Meanwhile, a calibration curve was made to examine whether the nomogram had a high accuracy of survival prediction.

### Biological enrichment analysis

Genes which *P* < 0.05 and the false discovery rate (FDR) q < 0.05 would be set as differentially expressed genes. The Gene Ontology (GO) function^18^ and Kyoto Encyclopedia of Genes and Genomes (KEGG)^19^ were used to analyze the differential pathway enrichment according to differentially expressed genes through the "clusterProfiler" R package. In addition, gene set enrichment analysis (GSEA) was utilized to identify the primary pathways in the two groups^20^.

### Immune infiltration of the model

The R package "GSEABase" was operated to find the differences between the high- and low- risk groups in immune function. Moreover, a histogram and a boxplot were drawn to visualize the differences in immune cell infiltration between the two groups.

### Tumor mutation burden and chemotherapy drug sensitivity

We employed waterfall plots to investigate the discrepancies in tumor mutation burden (TMB) between the two groups. Furthermore, we identified mutant genes and types of mutations which were occurred most frequently. According to the TMB score we calculated, we used a survival curve to analysis the differences in prognosis between the high- and low- TMB groups. In addition, we incorporated the TMB score and risk score to analyze the survival conditions of the HCC patients.

For further exploration of the clinical value of the model and drug sensitivity prediction, "oncoPredict" package was operated to calculate chemotherapy drug sensitivity. We downloaded data from Drug Sensitivity in Cancer (GDSC) and combined the data with transcriptomics data to estimate the differences in sensitivity between the high- and low- risk groups.

### Statistical analysis

All statistical analyses were performed using R 4.3.1 software and Perl packages were applied to deal with the data. Of all the processes, *P* < 0.05 was considered statistically significant.

### Ethical approval and consent to participate

The study complied with the principles of the Declaration of Helsinki. Access to the de-identifed linked dataset was obtained from the TCGA databases in accordance with the database policy. For analyses of de-identifed data from the TCGA databases, institutional review board approval and informed consent were not required.

## Results

**Figure 1:**
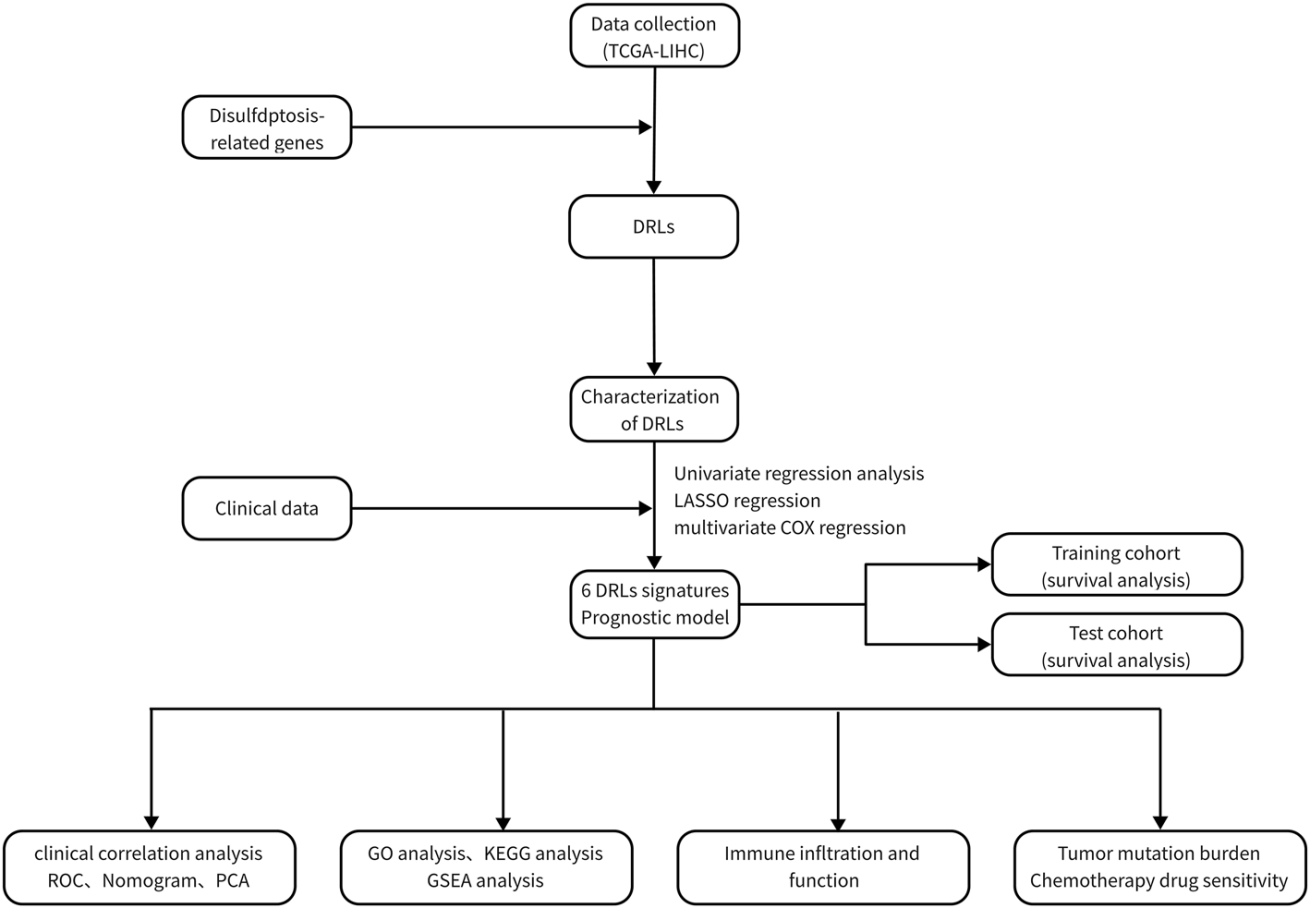
The overall study progress.

The overall study progress is shown in Fig. 1.

### Identification of disulfidptosis-related LncRNAs (DRLs) in HCC

**Figure 2:**
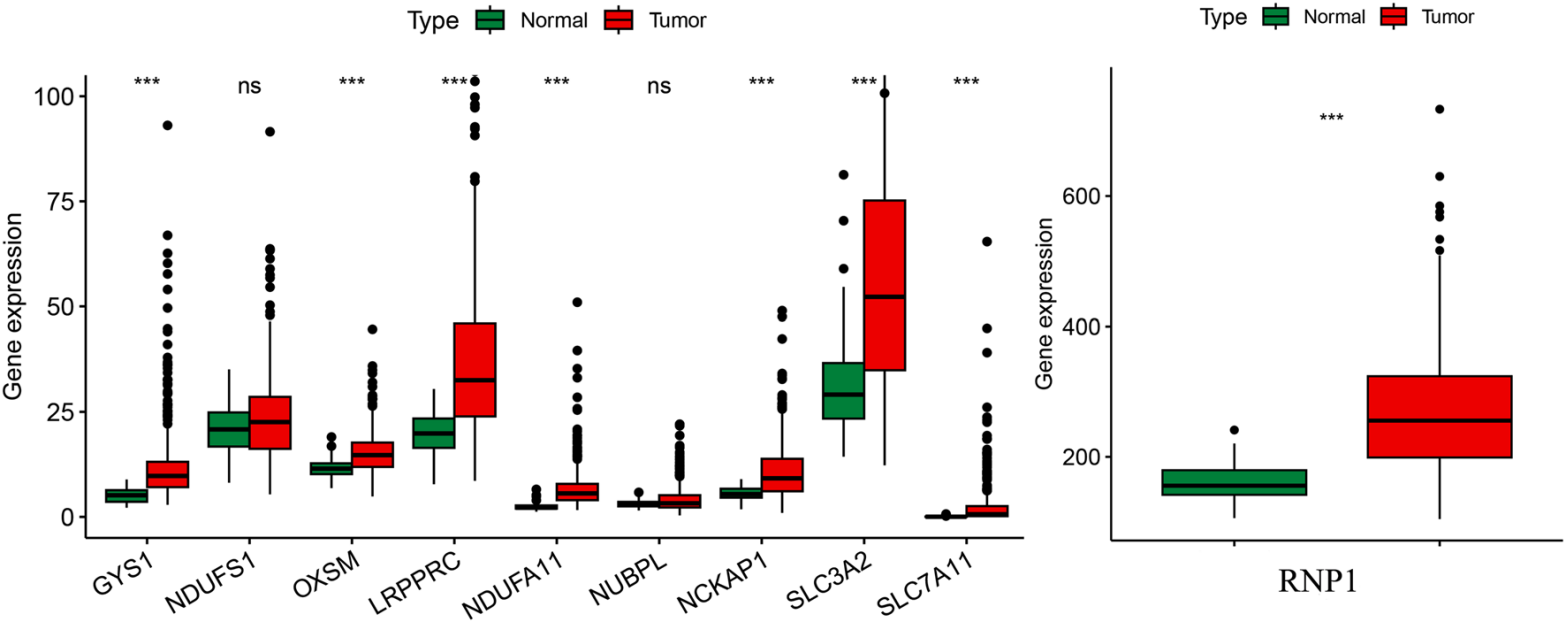
Differences in the expression of disulfdptosis-related genes between tumor tissues and normal tissues.

**Figure 3:**
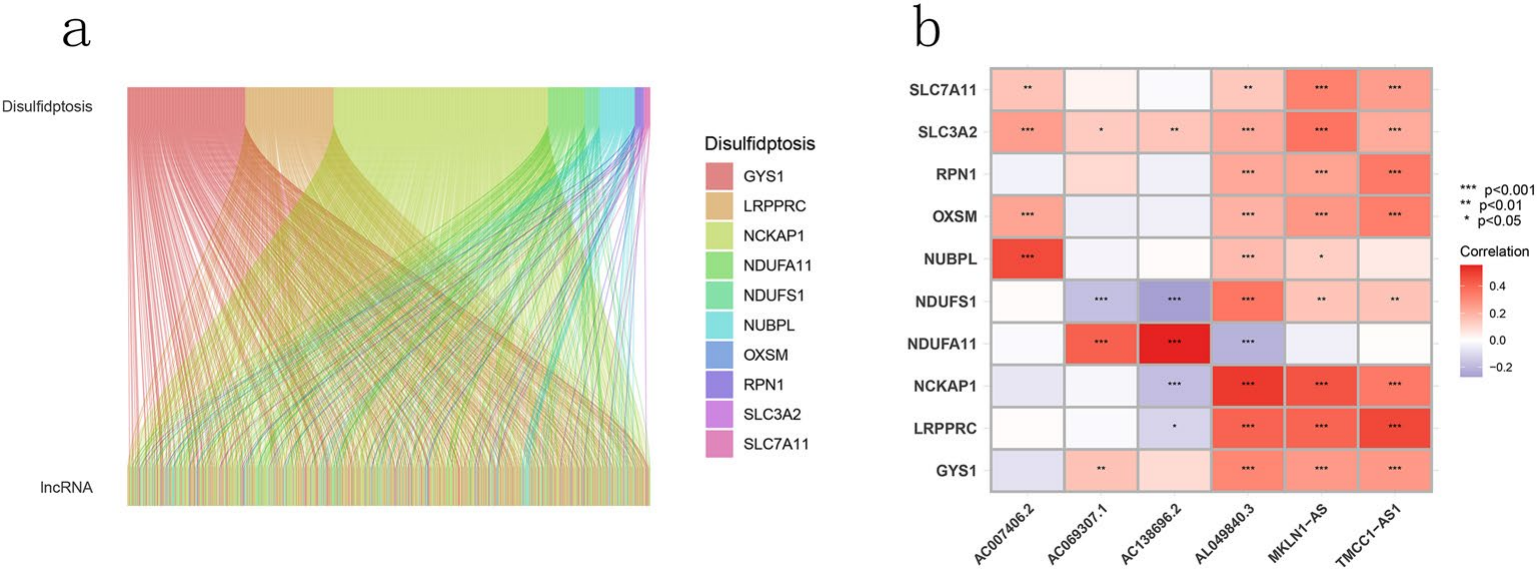
Identification of the signature of DRLs. (**a**) Sankey diagram of lncRNAs co-expressed with disulfdptosis-related genes. (**b**) Co-expression relationships between disulfdptosis-related lncRNA and disulfdptosis-related genes.

The genes GYS1, NDUFS1, OXSM, LRPPRC, NDUFA11, NUBPL, NCKAP1, RPN1, SLC3A2 and SLC7A11 were regarded as disulfdptosis-related genes according to pervious study. All the genes were highly expressed in tumor samples and except for the genes NDUFS1 and NUBPL, other genes showed apparent differences between the two groups (Fig. 2). A Sankey diagram was constructed to visualize the correlation between disulfdptosis-related genes and lncRNAs(Fig. 3a). As seen in the figure, NCKAP1 and GYS1 have the most connections with lncRNAs.

### Prognostic model and characterization of model

**Figure 4:**
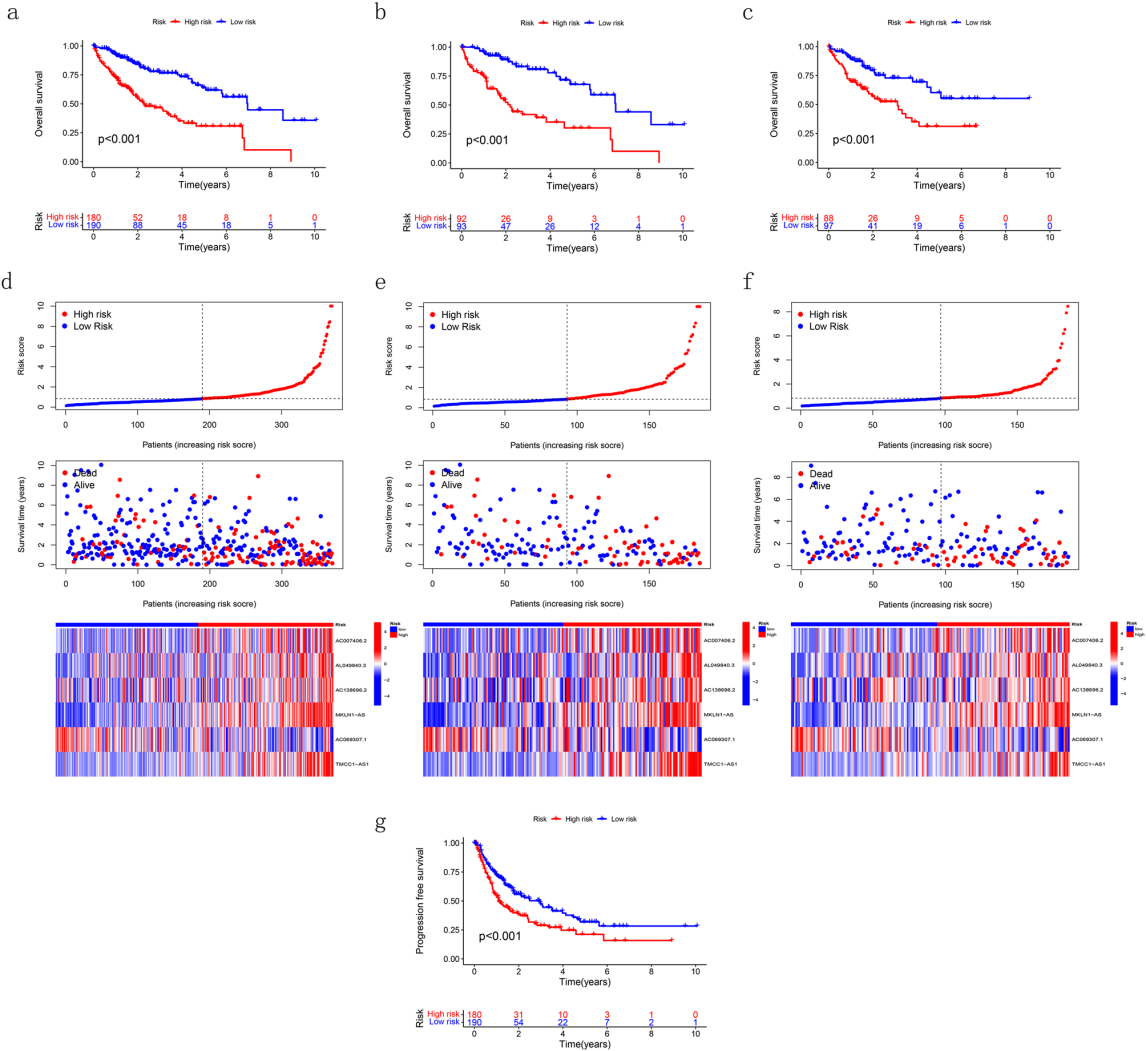
Characterizations of the DRLs model. (**a**–**c**) The OS of TCGA cohort, the training cohort, and the test cohort. (**d**–**f**) Survival curves between high- and low- risk groups in TCGA cohort, the training cohort and the test cohort. (**g**) The PFS survival analysis in TCGA cohort.

After combining transcriptomic and clinical data from TCGA, we randomly divided the samples into training and validation groups at a ratio of 1:1 and there was no significant difference in clinical characteristics between the two groups (Table 1). 88 DRLs were screened by using univariate Cox regression analysis. After that, the LASSO regression analysis was applied to find candidate DRLs. Finally, 6 DRLs were filtered to construct a prognostic model, including AC007406.2, AL049840.3, AC138696.2, MKLN1-AS, AC069307.1 and TMCC1-AS1. Among all the genes, AC007406.2, AL049840.3, AC138696.2, MKLN1-AS and TMCC1-AS1 were considered as positive factors for prognosis while AC069307.1 was considered as negative factor. The calculation formula of risk score was as follows: Risk-Score = [Expression level of AC007406.2 ∗ (0.17) + Expression level of AL049840.3 ∗ (0.63) + Expression level of AC138696.2 ∗ (0.27) + Expression level of MKLN1-AS ∗ (0.74)- Expression level of AC069307.1 ∗ (0.80) + Expression level of TMCC1-AS1 ∗ (0.79)]. In addition, co-expression analysis was performed between disulfdptosis-related genes and DRLs and the results are shown in Fig. 3b. Based on the formula, we divided the patients into high- and low- risk groups by the median risk score threshold (The median risk score threshold was 0.834941555). We used several prognostic analyses to assess the predictive value of the model. According to the OS and PFS curves, we identified that patients in high-risk group had a worse prognosis than those in the low-risk group (Fig. 4a–c,g). Meanwhile, survival curves were used to evaluate the risk of death between the two groups (Fig. 4d–f). As shown in the figures, the high-risk group had a superior risk of death compared to the low-risk group.

**Table 1:** Clinical characteristics between the two groups.

Characteristics	Total(n = 370)	Train(n = 185)	Test(n = 185)	*p* Value	
Age	< = 65	232(62.7%)	121(65.41%)	111(60%)	0.333
	> 65	138(37.3%)	64(34.59%)	74(40%)	
Gender	Male	249(67.3%)	123(66.49%)	126(68.11%)	0.825
	Female	121(32.7%)	62(33.51%)	59(31.89%)	
Grade	G1	55(14.86%)	25(13.51%)	30(16.22%)	0.807
	G2	177(47.84%)	86(46.49%)	91(49.19%)	
	G3	121(32.7%)	64(34.59%)	57(30.81%)	
	G4	12(3.24%)	6(3.24%)	6(3.24%)	
	unknow	5(1.35%)	4(2.16%)	1(0.54%)	
Stage	Stage I	171(46.22%)	84(45.41%)	87(47.03%)	0.16
	Stage II	85(22.97%)	43(23.24%)	42(22.7%)	
	Stage III	85(22.97%)	41(22.16%)	44(23.78%)	
	Stage IV	5(1.35%)	5(2.7%)	0(0%)	
	unknow	24(6.49%)	12(6.49%)	12(6.49%)	
T stage	T1	181(48.92%)	88(47.57%)	93(50.27%)	0.552
	T2	93(25.14%)	46(24.86%)	47(25.41%)	
	T3	80(21.62%)	39(21.08%)	41(22.16%)	
	T4	13(3.51%)	9(4.86%)	4(2.16%)	
	unknow	3(0.81%)	3(1.62%)	0(0%)	
M stage	M0	266(71.89%)	129(69.73%)	137(74.05%)	0.123
	M1	4(1.08%)	4(2.16%)	0(0%)	
	unknow	100(27.03%)	52(28.11%)	48(25.95%)	
N stage	N0	252(68.11%)	125(67.57%)	127(68.65%)	1
	N1	4(1.08%)	2(1.08%)	2(1.08%)	
	unknow	114(30.81%)	58(31.35%)	56(30.27%)

### Model predictive value assessment

**Figure 5:**
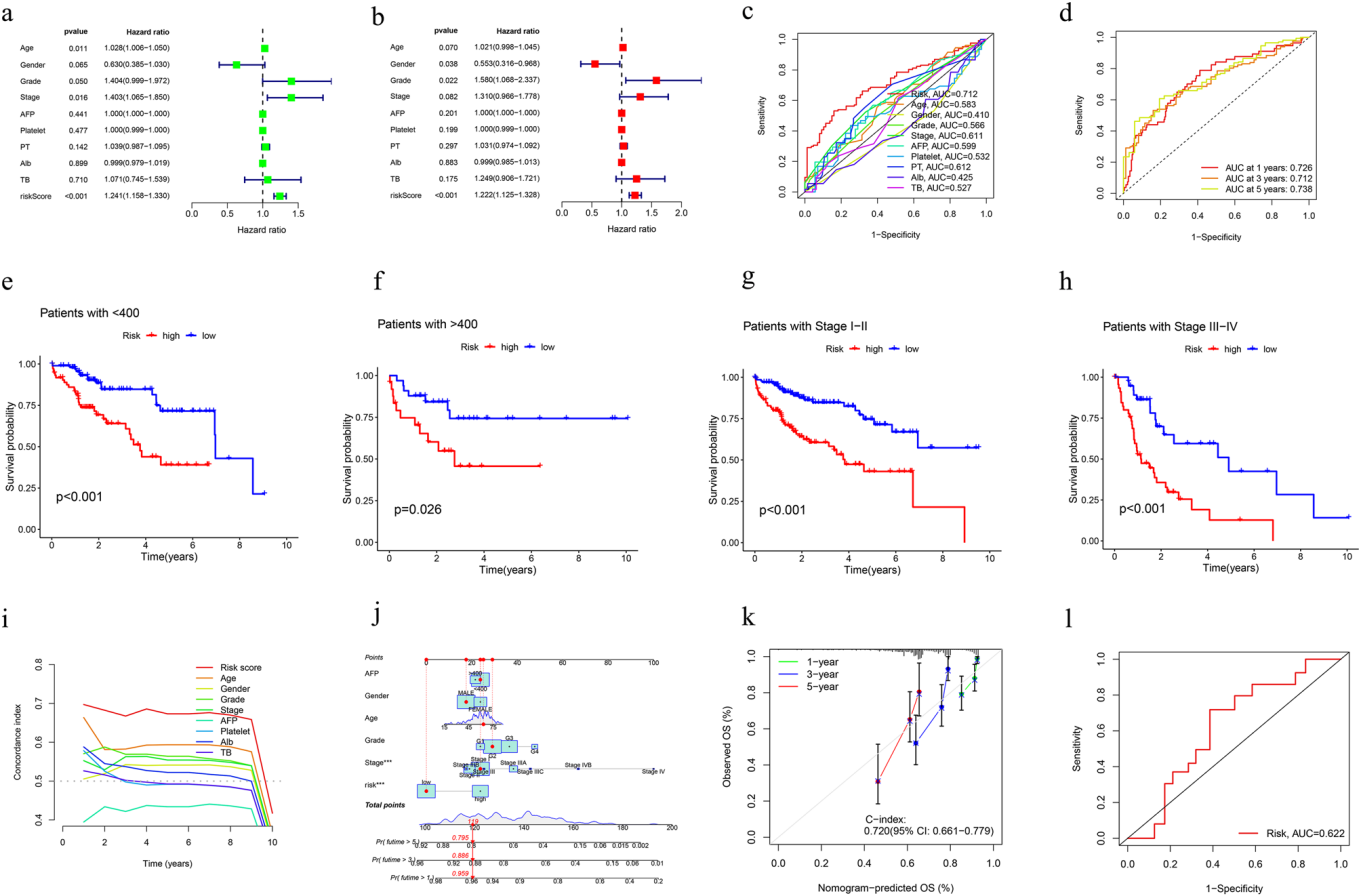
Validation of the disulfdptosis-related lncRNA signature. (**a**, **b**) Assessment of the independency of the model by univariate and multivariate Cox regression analyses (**c**) The AUC showed that risk score was an independent predictor compared with other clinical characteristics. (**d**) The predictive value in the OS of 1-, 3-, 5 years. (**e**, **f**) Survival curves of patients with early stage or advanced stage. (**g**, **h**) Survival curves of patients with Low and high value of AFP. (**i**) The C-Index of risk score was higher than other clinicopathological signatures. (**j**) The nomogram of DRLs model combined with other clinical features. (**k**) The calibration curve of the nomogram. (**l**) The ROC curve of validation cohort.

**Figure 6:**
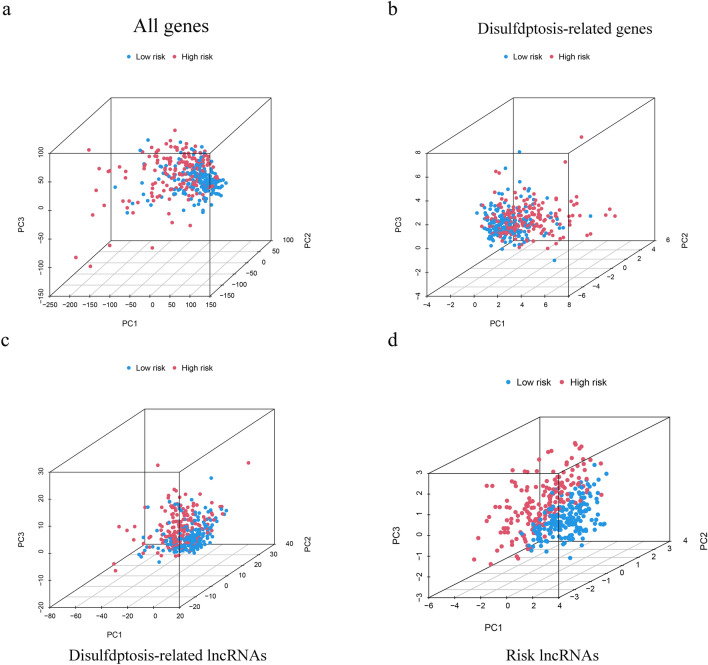
The outcomes of PCA in different gene sets.

By integrating clinical characteristics (including age, sex, tumor grade, tumor stage, AFP, TB, Alb, platelet and PT), we adopted univariate and multivariate Cox regression analyses to assess the independence of the model (Fig. 5a,b). The results suggested that risk score could serve as an independent prognostic factor in univariate Cox regression analysis and was also independent of other clinical traits according to multivariate Cox regression. Moreover, the ROC curve indicated that the model had a more accurate predictive ability than other clinical characteristics, with an area under curve (AUC) of 0.712 (Fig. 5c). As for the predictive value of survival, the AUCs of 1-year, 3-year and 5-year survival were 0.726, 0.712 and 0.738 (Fig. 5d). A c-index curve was plotted to further verify the predictive value, with c-index values greater than those of other traits (Fig. 5i). Furthermore, we drew a nomogram to provide a convenient and visualizable tool for prediction (Fig. 5j). With the growth of the nomscore, the survival rates of patients gradually decreased and the consistency of the predictions and actual measured results of the nomogram was validated by a calibration curve (Fig. 5k). Through dividing the clinical characteristics stage and the value of AFP into two groups, we found that whether in the early and advanced stage or low and high value of AFP, the low-risk group showed a better prognosis than high-risk group (Fig. 5e–h). As for the validation cohort, the AUC is 0.622 (Fig. 5l). The outcomes of PCA indicated that the DRL model had a better ability of risk cutoff compared with other gene sets (Fig. 6 a-d).

### Biological functional analysis

**Figure 7:**
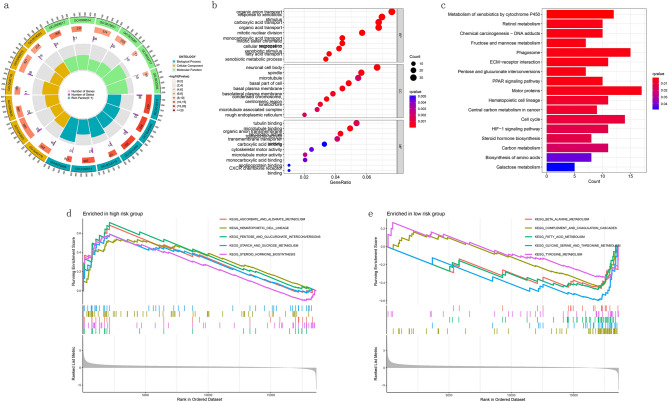
GO, KEGG and GSEA analysis. (**a**) The circle diagram of GO analysis. (**b**) The bubble diagram of GO analysis. (**c**) The barplot diagram of KEGG analysis. (**d**) GSEA analysis of the high-risk group. (**e**) GSEA analysis of the low-risk group.

According to a standard of fdr < 0.5, we investigated the differentially expressed genes between high- and low- risk groups. We operated GO and KEGG analyses and the results revealed the genes were primarily enriched in organic anion transport and motor proteins (Fig. 7a–c). In addition, GSEA analysis showed that the genes in the high-risk group were enriched mainly in pentose and glucuronate interconversions and ascorbate and aldarate metabolism (Fig. 7d). While in the low-risk group, they were functionally enriched mainly in glycine and theronine metabolism and fatty acid metabolism (Fig. 7e).

### Immune cells infiltration analysis

**Figure 8:**
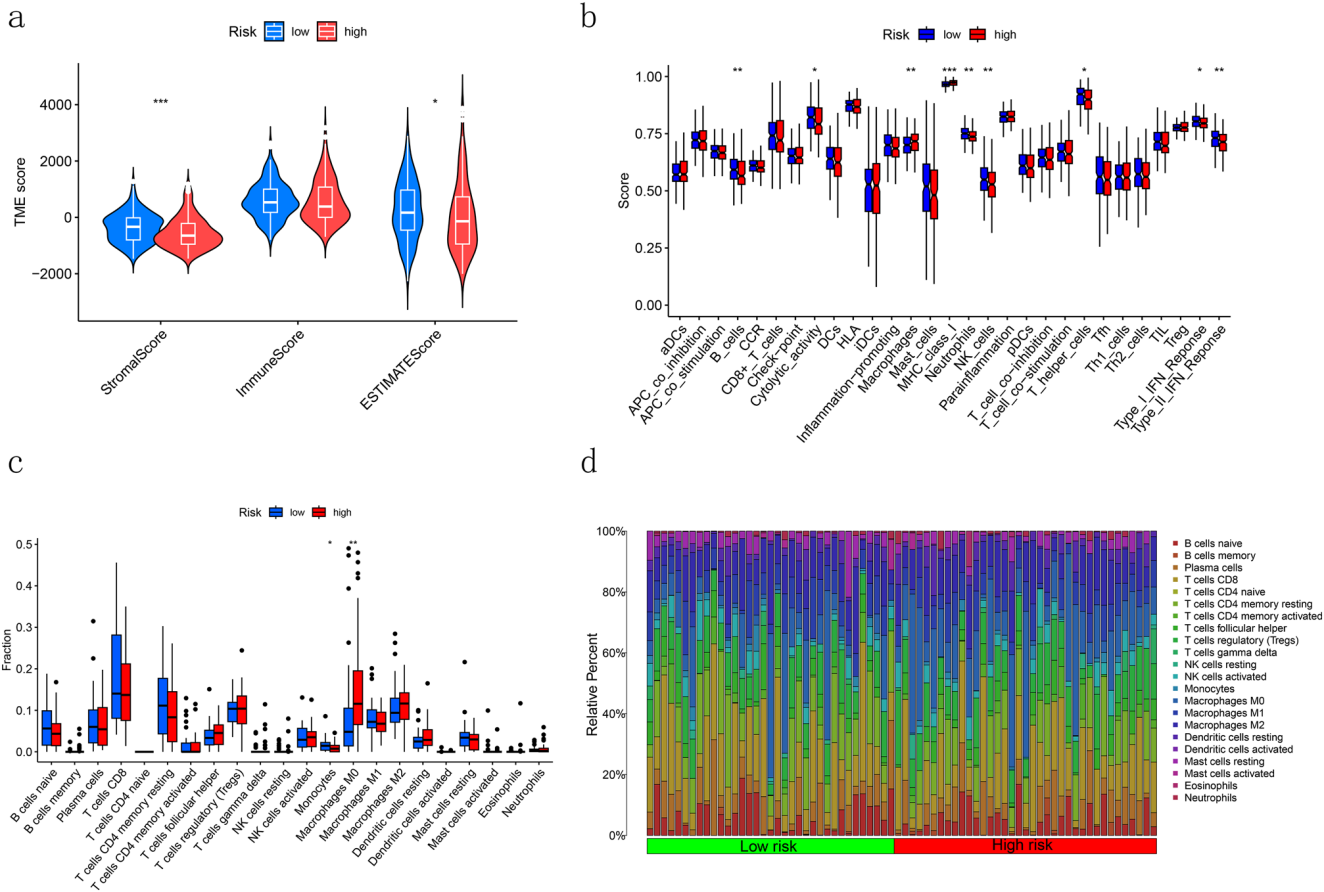
Immune infiltration analysis of the signature. (**a**) Stromal, immune, and ESTIMATE scores comparison between high- and low-risk group. (**b**) Differences in immune function between high- and low-risk groups. (**c**) Differences in immune cells infiltration between high- and low-risk group. (**d**) The barplot of differences in immune function between high- and low-risk groups.

Based on the TIME analysis, patients in high-risk group showed higher stromal and ESTIMATE scores than those in the low-risk group which indicating that tumor invasion and metastasis would be more likely to appear in the high-risk group(Fig. 8a).We evaluated the immune function between high- and low-risk groups and the results were presented in Fig. 8b. R package "Cibersoft" was used to analyze the difference in immune cell infiltration between two groups (Fig. 8c). The specific quantization of the difference was shown in Fig. 8d, suggesting that the low-risk group was enriched in several immune functions (B-cells, Neutrophils, NK-cells, T-helper cells, Type-I IFN response and Type-II IFN response) while the high-risk group was enriched mainly in MHC class-I and Macrophages.

### Tumor mutational burden

**Figure 9:**
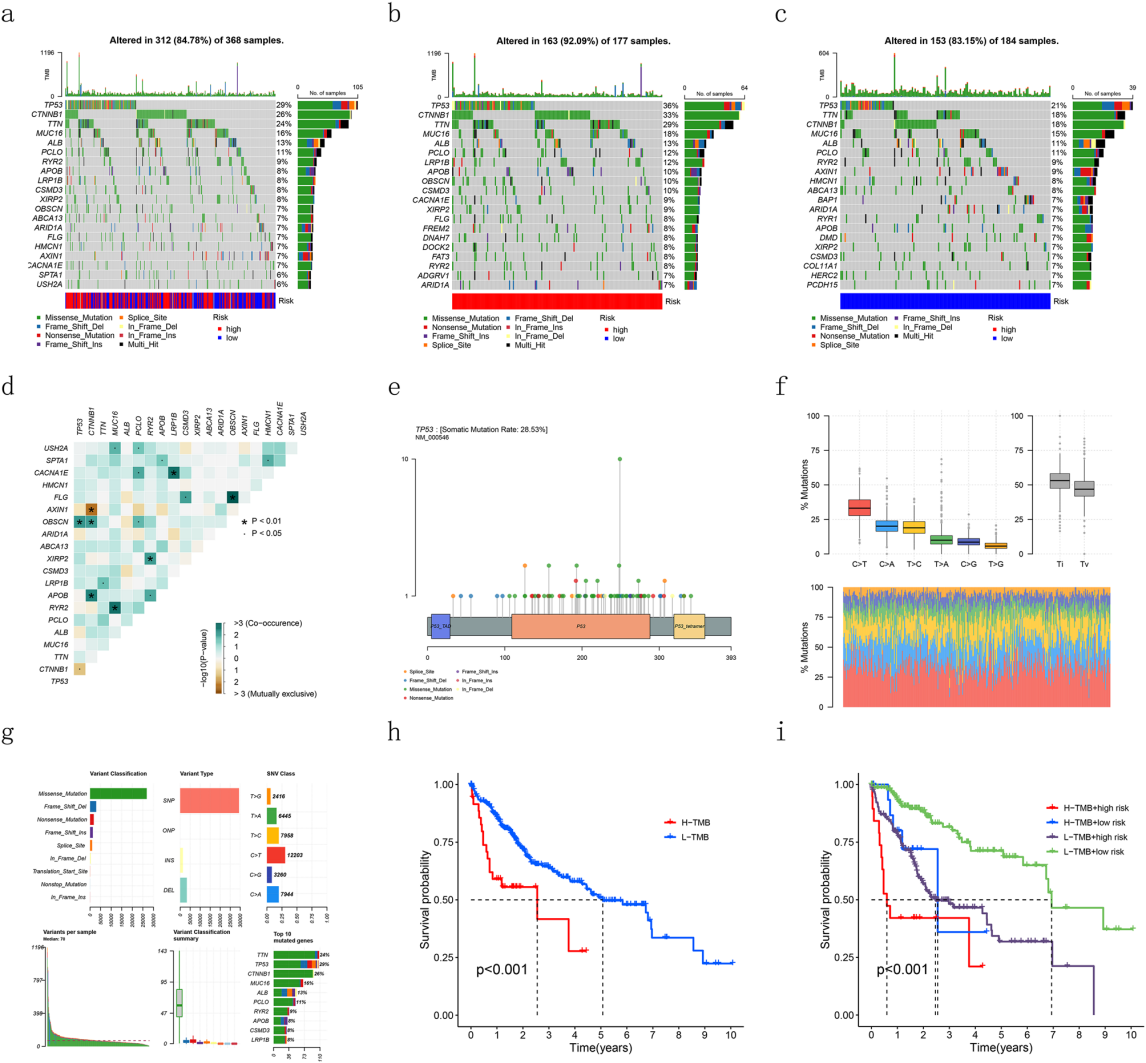
Relationship between DRLs model and tumor mutation burden. (**a**–**c**) Waterfall plots of somatic mutation characteristics in TCGA cohort, the training cohort and the test cohort. (**d**) Correlation analysis among the genes which mutated most. (**e**)The lollipop of gene TP53. (**f**, **g**) The identifications of the classifications of mutation. (**h**)The survival curve between high- and low-TMB groups. (**i**) K–M survival curves between the four groups.

Of all the samples, 20 genes including TP53, CTNNB1, TNN, MUC16, ALB, PCLO et al. were mutated most frequently (Fig. 9a). We operated the same analysis in both training cohort and validation cohort (Fig. 9b,c). In addition, we conducted a correlation analysis among the genes which mutated most (Fig. 9d). As for the gene TP53, we drew a lollipop to explore the frequency of mutation, the genetic locus which mutated most and the type of mutations (Fig. 9e). Results showed TP53 had a probability of 29% to mutate and missense mutation was the type of mutation occurred most frequently. Moreover, of all the classifications of mutations, missense mutation occurred most often, and single nucleotide polymorphisms (SNVs) were the most frequent type. Among all the types of SNV, transitions occurred more frequently than transversions, and conversion of cytosine to thymine had the most frequent occurrence (Fig. 9f,g). To assess the predictive value of the TMB score, we plotted a survival curve and the results showed that patients with low-TMB would have a better prognosis (Fig. 9h). The same results appeared in the survival curve when incorporating TMB and risk score. The low-TMB and low-risk groups had the best prognosis among all the groups (Fig. 9i).

### Prediction of chemotherapy drug sensitivity

**Figure 10:**
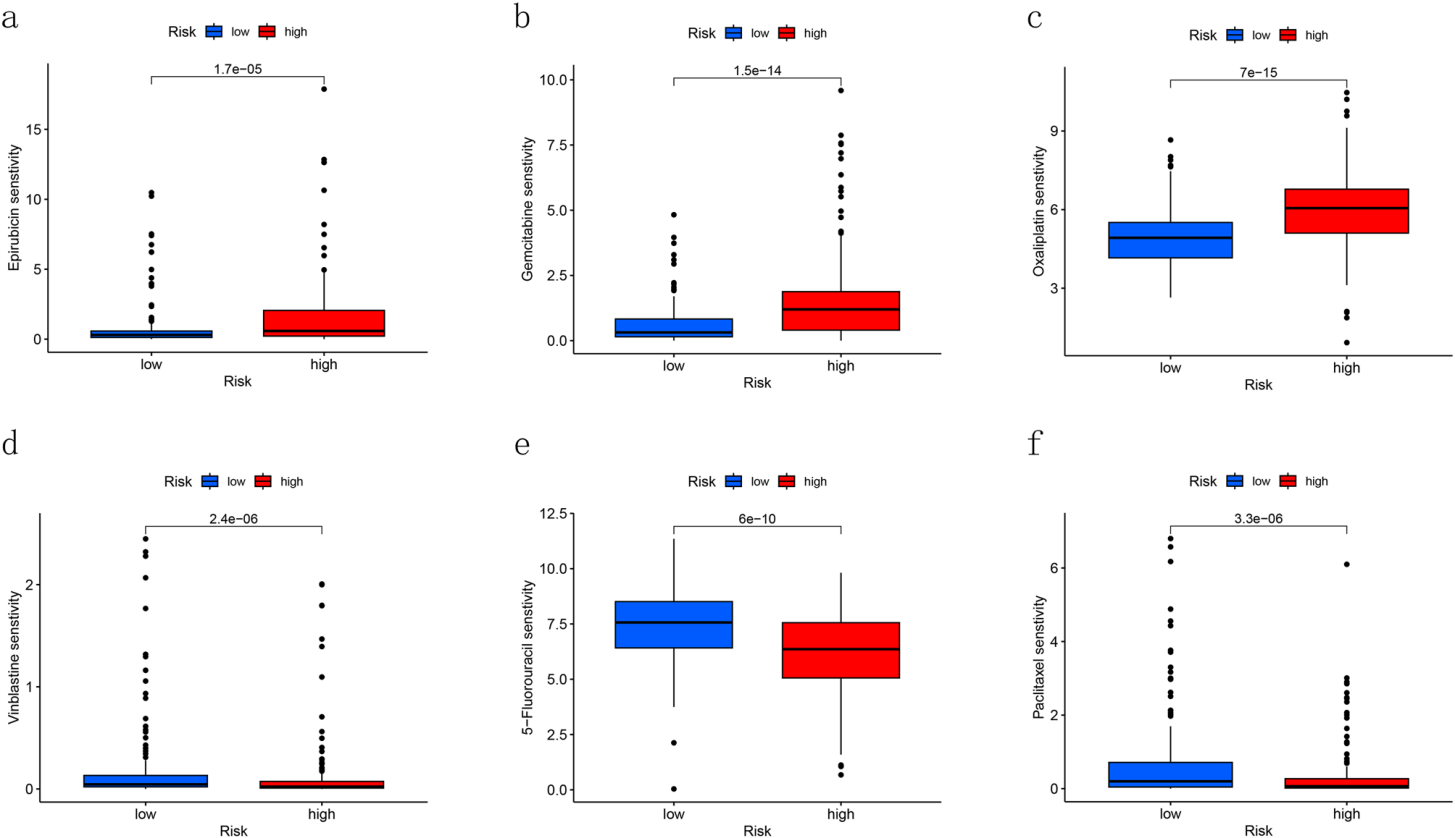
Chemotherapy drug sensitivity prediction for DRLs model. (**a**–**c**) Drugs which were more sensitive to patients in high-risk group. (**d**–**f**) Drugs which were more sensitive to patients in low-risk group.

Chemotherapy is one of the most considerable treatments for patients with HCC. We calculated the sensitivity of a series of chemotherapy drugs in high- and low- risk groups by using GDSC database. According to the results, epirubicin, gemcitabine and oxaliplatin were more sensitive to patients in high-risk group while vinblastine, paclitaxel and 5-fluorouracil seemed to be sensitive to low-risk group patients (Fig. 10a–f).

## Discussion

Liver cancer had been a major public health problem for many years all over the worlds. There are a large number of patients with hepatitis B, which causes HCC most in China. In the past 10 to 20 years, more and more researches had focused on the mechanisms of cancer occurrence and development. Therefore, targeted therapy and immunotherapy had become increasingly important with the development of molecular biology. In recent years, several prognostic models based on autophagy-related genes^21^, ferroptosis-related genes^22^, cuproptosis-related genes^23^, pyroptosis-related genes^24^ have been constructed to predict the prognosis of different cancers. Not only mRNAs but also lncRNAs had been used to establish prognostic models for predicting prognosis^25–28^. Disulfidptosis is a novel type of cell death. Rather than other types of regulated cell death, disulfidptosis would not be suppressed by cell death inhibitors, which would provide a new thread for cancer therapy.

To the best of our knowledge, there is no research on constructing disulfdptosis-related lncRNAs in HCC at present. In the present study, we discovered 746 lncRNAs, which had apparently correlationships with disulfdptosis-related genes. After a series of processes of dimensionality reduction, 6 disulfdptosis-related lncRNAs had been screened to construct a prognostic model, including AC007406.2, AL049840.3, AC138696.2, MKLN1-AS, AC069307.1 and TMCC1-AS. The DRL prognostic model showed an excellent ability of prognosis prediction according to the C-index and ROC curves. Among the six lncRNAs, several genes have been identified to be associated with tumor progression. For example, MKLN1-AS is upregulated in HCC tissues and promotes the proliferation and epithelial-mesenchymal transition (EMT) of HCC^29^. Chen found that TMCC1-AS could promote cell proliferation and migration in HCC^30^. Both articles showed the same results as our study. On the basis of the algorithm of the model, the risk score was calculated to separate the patients into two groups and the low-risk group had a better prognosis according to the OS and PFS curves. Further validation was conducted to evaluate the accuracy, independence, sensitivity, and specificity of the model’s value of prediction. Independent prognostic analysis suggested that the model was an independent prognostic factor for HCC patients. The ROC and C-index curves identified that the accuracy, sensitivity and specificity of the model were superior to other clinical characteristics. Such results demonstrated that the model could be a novel method for prognostic prediction of HCC. The results of PCA showed that the model had an excellent ability to distinguish low- and high-risk patients. GSEA showed that in high-risk group, the genes were mainly enriched in glycometabolism steroid metabolism. While in the low-risk group, they were mainly enriched in amino acid metabolism and lipid metabolism.

The immune environment is of vital importance to the tumor microenvironment. Our findings revealed that the immune functions of high-risk group were enriched mainly in MHC class-I and Macrophages. As for the immune cells infiltration, there was a better enrichment of CD4 + T cells and CD8 + T cells in low-risk group and the results were the same as a previous study in which CD8 + T cells may be related to superior prognosis^31^.

TMB, which is related to immune checkpoint inhibitors (ICIs) and prognosis, has become more and more important for cancer therapy. Our study showed that there was no significant difference between high- and low-risk groups in TMB. However, by integrating TMB and risk-score, we found patients with low-TMB and low-risk had the best prognosis while the prognosis of patients with high-TMB and high-risk was worst. Gene TP53 showed the highest frequency of mutation in both groups, with a proportion of 29% of all the patients. TP53 was identified to be related to colorectal cancer liver metastasis^32^. Similarly, Tornesello^33^ found the mutation of TP53 could promote the progress of liver cancer. In addition, we calculated the sensitivity of several chemotherapy drugs. Epirubicin, gemcitabine, oxaliplatin, vinblastine, paclitaxel and 5-fluorouracil are common chemotherapy drugs for patients with HCC. In our study, we found epirubicin, gemcitabine and oxaliplatin were sensitive to patients in high-risk group and in contrast, vinblastine, paclitaxel and 5-fluorouracil were sensitive to patients in low-risk group.

There are several deficiencies that need to be mentioned. First, this study is theoretically feasible because we only analyzed the data from TCGA and we did not verify it in vivo or vitro. Second, disulfidptosis is a new type of RCD which means there are not enough researches on it and the biological significance of six long stranded non coding RNAs is not yet clear, and it is possible that they affect prognosis through a mechanism completely unrelated to disulfide deficiency. Therefore, the disulfidptosis-related we used may not contain all genes which would lead to imprecise results. Third, external validation may be taken to further evaluate the prognostic value of the 6-DRLs model. Thus, further studies and more experiments should be carried out to validate the model and biomarker.

## Conclusion

We constructed a 6 disulfidptosis -related lncRNAs prognostic model and based on the model, we separated HCC patients into two groups. There were significant differences between two groups in survival outcomes. In addition, the independence, accuracy, and specificity of the model had been validated. In summary, this model could be a therapeutic biomarker for the treatment and prognosis of HCC patients.

## Data Availability

Data used to support the fndings of this study are available from the corresponding authors upon request.
